# Azithromycin enhances anticancer activity of TRAIL by inhibiting autophagy and up-regulating the protein levels of DR4/5 in colon cancer cells in vitro and in vivo

**DOI:** 10.1186/s40880-018-0309-9

**Published:** 2018-07-03

**Authors:** Xinran Qiao, Xiaofei Wang, Yue Shang, Yi Li, Shu-zhen Chen

**Affiliations:** Institute of Medicinal Biotechnology, Chinese Academy of Medical Sciences & Peking Union Medical College, Beijing, 100050 P.R. China

**Keywords:** Azithromycin, TRAIL, Apoptosis, Autophagy, Colon cancer

## Abstract

**Background:**

Azithromycin is a member of macrolide antibiotics, and has been reported to inhibit the proliferation of cancer cells. However, the underlying mechanisms are not been fully elucidated. Tumor necrosis factor-related apoptosis-inducing ligand (TRAIL) selectively targets tumor cells without damaging healthy cells. In the present study, we examined whether azithromycin is synergistic with TRAIL, and if so, the underlying mechanisms in colon cancers.

**Methods:**

HCT-116, SW480, SW620 and DiFi cells were treated with azithromycin, purified TRAIL, or their combination. A sulforhoddamine B assay was used to examine cell survival. Apoptosis was examined using annexin V-FITC/PI staining, and autophagy was observed by acridine orange staining. Western blot analysis was used to detect protein expression levels. In mechanistic experiments, siRNAs were used to knockdown death receptors (DR4, DR5) and LC-3B. The anticancer effect of azithromycin and TRAIL was also examined in BALB/c nude mice carrying HCT-116 xenografts.

**Results:**

Azithromycin decreased the proliferation of HCT-116 and SW480 cells in a dose-dependent manner. Combination of azithromycin and TRAIL inhibited tumor growth in a manner that could not be explained by additive effects. Azithromycin increased the expressions of DR4, DR5, p62 and LC-3B proteins and potentiated induction of apoptosis by TRAIL. Knockdown of DR4 and DR5 with siRNAs increased cell survival rate and decreased the expression of cleaved-PARP induced by the combination of azithromycin and TRAIL. LC-3B siRNA and CQ potentiated the anti-proliferation activity of TRAIL alone, and increased the expressions of DR4 and DR5.

**Conclusion:**

The synergistic antitumor effect of azithromycin and TRAIL mainly relies on the up-regulations of DR4 and DR5, which in turn result from LC-3B-involved autophagy inhibition.

**Electronic supplementary material:**

The online version of this article (10.1186/s40880-018-0309-9) contains supplementary material, which is available to authorized users.

## Background

Azithromycin is a macrolide antibiotic widely used to treat bacterial infection. Azithromycin accumulates and undergoes slow release in cells, especially in phagocytes, and thus has higher local concentration and longer half-life than older macrolides (i.e., erythromycin and clarithromycin) [1, 2]. Recent studies have indicated that azithromycin could produce potent anti-proliferation effect by inducing apoptosis in HeLa cells and SGC-7901 cancer cells [3]. Clinical studies have suggested promising efficacy of azithromycin in combination with paclitaxel and cisplatin in stage III–IV NSCLC patients [4]. In addition, it has been reported that antibiotics could affect tumor growth by targeting mitochondria and eradicating cancer stem cells [5]. Macrolide antibiotics (e.g., azithromycin, clarithromycin and erythromycin) sensitize cancer cells to the apoptotic effect of bortezomib and EGFR-TKI by blocking autophagy flux, but the targets of azithromycin in the apoptosis pathway remain unknown [6, 7]. Preclinical and clinical data suggest that clarithromycin in combination with conventional chemotherapeutic agents could produce robust antitumor activities [8, 9]. However, few studies have explored potential action of azithromycin.

Tumor necrosis factor-related apoptosis-inducing ligand (TRAIL) is a member of the tumor necrosis factor superfamily, and considered a promising tumor therapeutic agent since it selectively targets tumor cells without producing cytotoxicity in healthy cells. TRAIL interacts with, and causes the clustering of death receptors (DR) 4 and 5, and induces the assembly of DISC and the activation of a series of downstream caspase cascades [10]. This pathway is known as the extrinsic apoptosis pathway [10]. Interestingly, past studies have produced seemingly opposite effects: increasing the expression of DR4/5 is related to survival in colon cancer patients [11]. Primary human colon cancer cells and high-grade adenomas are sensitive to TRAIL and combinational chemotherapies (e.g., using chloroquine and shogaol with TRAIL) could inhibit the proliferation of colon cells more effectively [12–16].

In the present study, we examined whether azithromycin and TRAIL could produce synergistic effects in colon cancers. Potential action of autophagy inhibition and apoptosis in the interaction was also examined.

## Materials and methods

### Cells culture

A total of 4 human colon adenocarcinoma cell lines were used in the current study. SW480 and SW620 cells were obtained from the Cell Resource Center of the institute of Basic Medical Sciences (IBMS) of the Chinese Academy of Medical Sciences (CAMS) & Peking Union Medical College (PUMC) (Beijing, China), respectively, and cultured in Iscove’s Modified Dulbecco’s Medium and Eibovitz’s L-15 Medium (ThermoFisher Scientific, Waltham, MA, USA). HCT-116 cells were kept in our laboratory in RPMI-1640 medium (ThermoFisher Scientific). DiFi cell line was a gift from Professor Wang Zhen at our institute and cultured in Dulbecco’s Modified Eagle Medium: nutrient mixture F-12 (1:1) medium (ThermoFisher Scientific). For all cell lines, the culture medium was supplemented with 10% fetal bovine serum (Gibco, Carlsbad, California, USA), 100-U/mL penicillin and 100-µg/mL streptomycin (North China Pharmaceutical Inc, Beijing, China) at 37 °C in a 5% CO_2_ incubator.

### Reagents and antibodies

Azithromycin and *N*-acetylcysteine (NAC) were obtained from National Institutes for Food and Drug Control (Beijing, China). Azithromycin was dissolved in anhydrous ethanol; NAC was dissolved in distilled water. Anti-caspase-3, anti-Akt (pan), anti-p44/42 MAPK (Erk 1/2), anti-p38 MAPK, anti-PARP and p62 antibodies were purchased from Cell Signaling Technology (Danvers, MA, USA). Anti-LC3B antibody was purchased from Sigma (St. Louis, MO, USA). Azide-free anti-human CD261 (DR4) antibody was obtained from Diaclone (Besancon, France). Anti-DR5 antibody was obtained from ProSci (San Diego, California, USA). β-Actin (6G3) and GAPDH (1C4) monoclonal antibodies were purchased from AmeriBiopharma (Wilmington, Delaware, USA). Secondary antibodies included peroxidase-conjugated affiniPure goat anti-mouse IgG (H+L) and goat anti-rabbit IgG (H+L) (ZSGB-BIO, Beijing, China). Caspase inhibitor zVAD-fmk and RIP1 inhibitor necrostatin-1 were purchased from Selleck.cn (Houston, TX, USA). Chloroquine (CQ), acridine orange hemi (zinc chloride) salt (AO) and sulforhodamine B (SRB) were from Sigma.

### Expression and purification of TRAIL protein

Recombinant TRAIL was constructed in this laboratory and expressed in *P. pastoris*. TRAIL expressing strains were inoculated in 100-mL BMGY medium (100 mmol/L potassium phosphate buffer, pH 6.0, 1% yeast extract, 2% peptone, 1% glycerol, 1.34% YNB, 0.00004% biotin) in a shaking incubator at 30 °C for 36 h. Yeast cells were precipitated and re-suspended in 100-mL BMMY with added 1.5% methanol every 24 h, at 26 °C for 72 h. Supernatant was collected by centrifugation at 7800 × *g*, 4 °C for 15 min. TRAIL protein was purified with Ni^2+^ affinity chromatography (His Trap HP, GE Healthcare, Pittsburgh, Pennsylvania, USA). Protein concentration was examined using a BCA method.

### Cell survival assay

Cells were seeded in 96-well plates at 3 × 10^3^ cells/well in 100-µL culture medium. Twenty-four hour later, cells were exposed to test drugs (azithromycin and TRAIL of varying concentrations, and combination) for 24, 48, or 72 h prior to survival assay using a sulforhodamine B (SRB) method [17]. Cell survival was calculated relative to the control group.

### Western blot analysis

Cells were lysed with a lysis buffer (50 mmol/L Tris–HCl pH 8.0; 2% NP-40; 150 mmol/L NaCl; 0.2% SDS; 0.5% sodium deoxycholate) containing 1% protease inhibitor (Beyotime, Jiangsu, China) and 100-µmol/L phenylmethylsulfonyl fluoride (PMSF). The supernatant was collected after centrifugation at 13,000 × *g* for 15 min at 4 °C, prior to Western blotting analyses, as described previously [18].

### Apoptosis assay

Apoptosis was determined using an annexin V-FITC/PI apoptosis detection kit from DOJINDO (Shanghai, China). A schematic plot was used to display the results: the lower left quadrant represents live cells; the lower right and upper right quadrants represent early and late apoptotic cells, respectively; the upper left quadrant represents necrotic cells. Cell death refers to the sum of early and late apoptotic and necrotic cells.

### Acridine orange (AO) staining

HCT-116 and SW480 cells were plated into 6-well plates and treated with drugs for 24 h. Later, cells were washed by PBS twice and stained with 700 µL/well AO (1 µg/mL) for 15 min at 37 °C in the dark. Then, the cells were washed by PBS twice. Watching the images under a fluorescence microscope through a 490 nm band-pass excitation filter and a 515 nm long-pass barrier filter. The green color represented the nucleus, while the red represented the acidic vesicles.

### siRNA transfection

DR4 siRNA (sense: 5′-AACGAGATTCTGAGCAACGCA-3′, anti-sense: 3′-TTGCTCTAAGACTCGTTGCGT-5′), DR5 siRNA (sense: 5′-AAGACCCTTGTGCTCGTTGTC-3′, anti-sense: 3′-TTCTGGGAACACGAGCAACAG-5′), LC-3B siRNA (sense: 5′-GGTGTATGAGAGTGAGAAA-3′, anti-sense: 3′-CCACATACTCTCACACTTT-5′) and negative siRNA were purchased from Ruibo Biotechnology (Guangzhou, China) and dissolved in RNase-free water as a 20 µmol/L stock. Negative siRNA was designed by Ruibo biotechnology and belonged to scrambled control. Cells were transfected with siRNAs using the Ruibo FECT™ CP transfection kit, plated in 96-well or 6-well plates and incubated at 37 °C for 24 h. siRNAs were diluted in transfection reagent and incubated for 15 min at room temperature to allow the formation of transfection complexes prior to addition to the cells (final concentration: 30 nmol/L). Experiments with test drugs started 24 h after the transfection. Efficiency of transfection was verified with Western blotting.

### Colon cancer xenograft

All animal experiments were performed in accordance with relevant guidelines and regulations. Briefly, HCT-116 cells (1 × 10^7^ cells in 200-µL PBS) were injected into the right armpits of 6-week-old female BALB/c nude mice (SPF Biotechnology Co., Ltd., Beijing, China). At 21 days after the inoculation, tumors were removed and cut into 2 m × 2 m × 2 m prisms, and transplanted into the right flanks of other mice through a trocar. Seven days later, mice were randomized to receive azithromycin (50 mg/kg/day, via oral administration, for 3 consecutive days in a week) or TRAIL (10 mg/kg, via the tail vein, once a week). Tumor volumes and body weights were monitored once every 2 days. The tumor volume was calculated by the following formula: *V* = ab^2^/2 (a represents the length of the tumor and b represents the width). The animal experiment lasted for a total of 32 days. At the end of experiment, the tumors were removed and fixed with formalin to detect cell proliferation by immunohistochemistry.

### Ki-67

Tissue sections (5 µm) were quenched with 3% H_2_O_2_ for 10 min at room temperature after dewaxing and antigen retrieval in hot citrate buffer. After blocking with 5% BSA for 30 min at 37 °C, tissue sections were incubated with a monoclonal anti-Ki-67 nuclear antigen antibody (ZSGB-BIO) at 4 °C overnight. Immunostaining was assessed in 3 randomly selected fields under a microscope with a 200 × objective lens and photographed. Images were analyzed using a microscope-matched analytical software (Leica QWin Standard).

### Assessment of drug interaction

The mode of drug interaction was evaluated based on the coefficient of drug interaction (CDI), calculated as: CDI = AB/(A × B), where AB is the survival rate of the cells exposed to both agents and A or B is the survival rate of cells exposed to either agent alone. A CDI at < 1.0 indicates synergistic effect; a CDI at < 0.7 indicates strongly synergistic effect [17].

### Statistical analysis

All experiments were repeated for at least three times. Data are expressed as the mean ± SD, and analyzed with Student’s *t* test for independent samples. Statistical significance was set at *P* < 0.05.

## Results

### Azithromycin inhibited cell proliferation

**Fig. 1:**
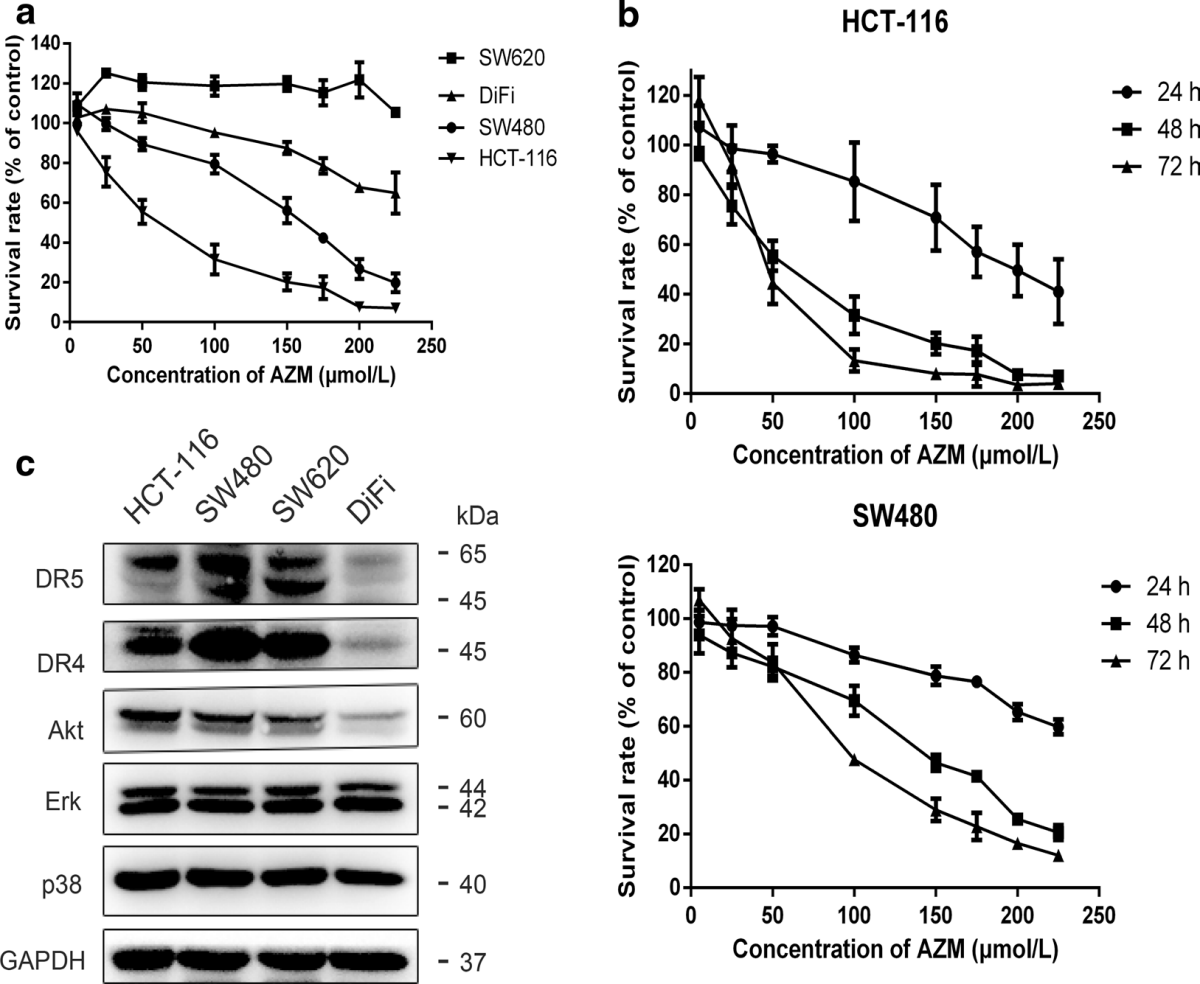
Azithromycin inhibits cell proliferation of colon cancer cell lines. **a** The viability of SW620, DiFi, SW480 and HCT-116 cells after 48 h-of treatment with azithromycin at various concentrations was assessed with the SRB assay. **b** The proliferative activity of HCT-116 and SW480 cells after treatment with azithromycin (5–225 µmol/L) for 24–72 h was assessed by the SRB assay. The survival rate was calculated as a ratio to the control group (untreated cells). Values represent the mean ± SD of three independent samples. Each experiment was repeated three times. **c** DR4/5 expression, Akt and MAPK pathways of colon cancer cell lines. HCT-116, SW480, SW620 and DiFi cells not receiving any treatment were used as controls. AZM represents azithromycin

In a pilot experiments with four colon cancer cell lines (SW620, DiFi, SW480 and HCT-116), only SW480 and HCT-116 cells were sensitive to azithromycin (Fig. 1a). Accordingly, subsequent experiments were conducted in SW480 and HCT-116 cells. Azithromycin inhibited cell proliferation in a dose- and time-dependent manner in both HCT-116 and SW480 cells (Fig. 1b). The half-inhibitory concentration (IC_50_) upon 48-h treatment was 63.19 ± 24.60 and 140.85 ± 32.81 µmol/L in HCT-116 and SW480 cells, respectively. Western blot analysis failed to showed differences of Erk and p38 MAPK expression among the four cell lines (Fig. 1c). In contrast, DR4/5 and Akt proteins in DiFi cells were much lower than in other cell lines, suggesting that DR4/5, MAPK and Akt signal pathways may be not related to azithromycin sensitivity in our experimental system.

### Azithromycin and TRAIL inhibit cell proliferation synergistically

**Fig. 2:**
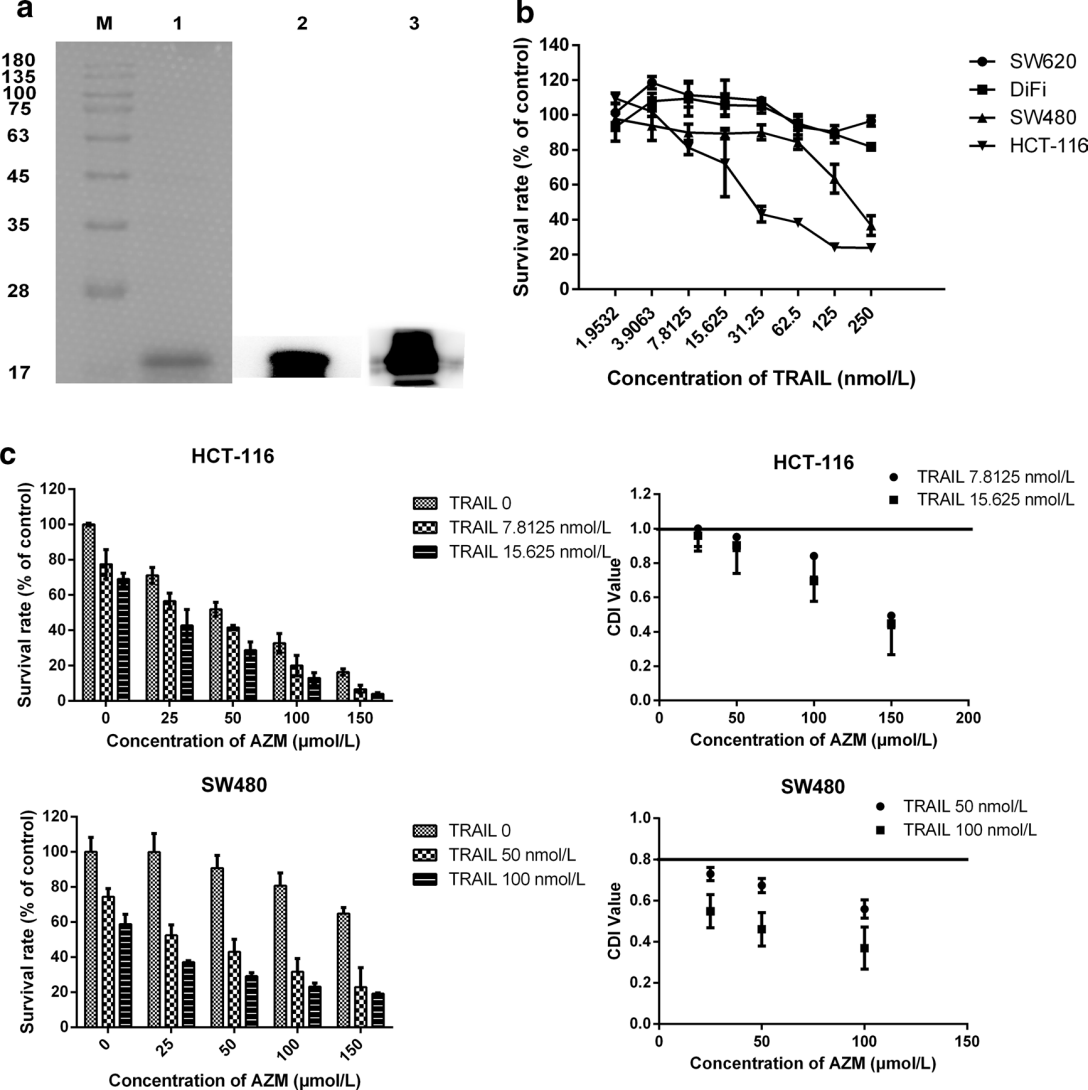
The anti-survival effect of TRAIL alone and the combination of azithromycin and TRAIL in colon cancer cells. **a** The purification of TRAIL protein. M represents marker; 1 represents TRAIL protein; 2 represents Western blot by His-tag antibody; 3 represents Western blot by anti-TRAIL antibody. **b** The proliferation of four types of colon cancer cells (SW620, DiFi, SW480 and HCT-116) treated with various concentrations of TRAIL for 24 h, as detected by a SRB assay. **c** The synergistic inhibitory effect of azithromycin and TRAIL on the proliferation of SW480 and HCT-116 cells. HCT-116 cells were treated with azithromycin (25–150 µmol/L) and TRAIL (7.8125 and 15.625 nmol/L) for 48 h; SW480 cells were treated with TRAIL at 50 and 100 nmol/L. The survival rate was calculated as a ratio to the control group (untreated cells). CDI value < 1 represents a synergistic effect and CDI < 0.7 indicates a robust synergistic effect. Values represent the mean ± SD of three independent samples. Each experiment was repeated three times. AZM represents azithromycin

The current study examined the potential interaction between azithromycin and TRAIL. Expression, purification and verification of TRAIL (using anti-His Tag and anti-TRAIL antibody) were illustrated in Fig. 2a. Although SW480 and HCT-116 cells were chose to subsequent experiments due to their sensitivity to azithromycin, we also needed to identify that if they were more sensitive to TRAIL than two other cells, so SRB assay was used to detect the anti-proliferation effects of TRAIL in four kinds of colon cancer cells and the results showed that TRAIL decreased the cell viability of SW480 and HCT-116 cells in a dose-dependent manner (Fig. 2b). Next, we exposed these four kinds of cells to TRAIL alone, azithromycin alone, or the drug combination for 48 h. The results showed that there was no synergism between TRAIL alone, azithromycin in SW620 and DiFi cells (data not shown). However, the combination of azithromycin at 25–150 µmol/L with TRAIL (7.8125 and 16.625 nmol/L) was much more effective in reducing HCT-116 and SW480 cell proliferation than either single agent alone (Fig. 2c). The CDI was 0.5–1 in HCT-116 cells and 0.3–0.7 in SW480 cells.

### Azithromycin enhanced TRAIL-induced cell death in HCT-116 and SW480 cells

**Fig. 3:**
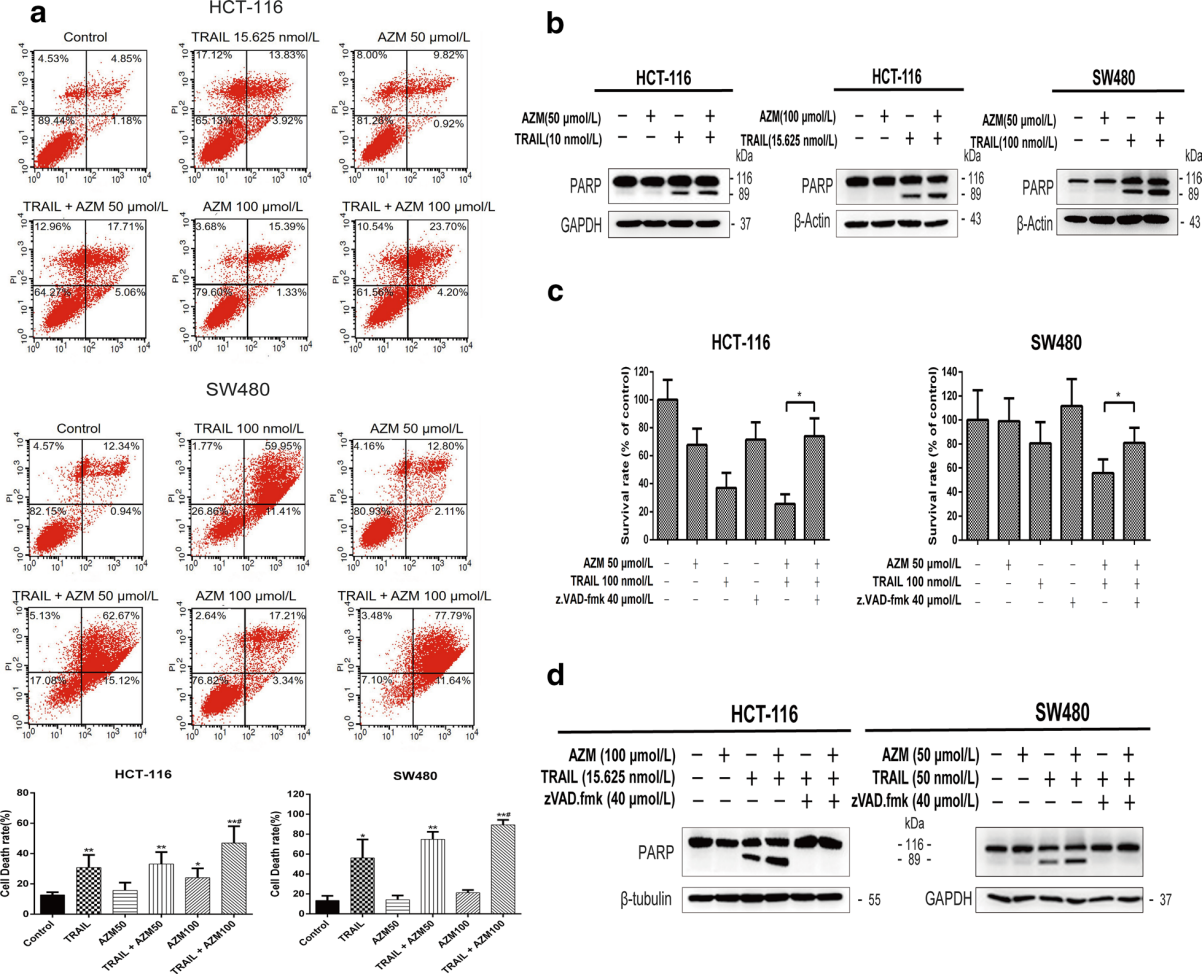
Azithromycin enhances TRAIL-induced cell death in HCT-116 and SW480 cells. **a** HCT-116 and SW480 cells were treated with azithromycin (50 and 100 µmol/L) and TRAIL (15.625 or 100 nmol/L, respectively) for 24 h. Apoptosis was detected by annexin V-FITC/PI staining and flow cytometry analysis. The cell death rate is the sum of the percentages of early, late apoptotic and necrotic cells. ^*^P < 0.05 and ^**^P < 0.01 vs. control. ^#^*P *< 0.05 and ^##^*P *< 0.01 vs. TRAIL alone. **b** Cell lines were exposed to azithromycin (50 or 100 µmol/L) and/or TRAIL (10, 15.625 or 100 nmol/L) for 10 h. The expression levels of proteins were detected by Western blot analysis. **c** zVAD.fmk reversed the synergistic inhibitory effect of azithromycin and TRAIL. Both cell lines were treated with azithromycin (50 µmol/L), TRAIL (100 nmol/L) and zVAD.fmk (40 µmol/L) (zVAD.fmk pretreatment for 0.5 h) for 48 h. ^*^P < 0.05. **d** zVAD.fmk also significantly reduced the expression level of cleaved-PARP. Cell lines were exposed to azithromycin (50 or 100 µmol/L), TRAIL (15.625 or 50 nmol/L) and zVAD.fmk (40 µmol/L) (zVAD.fmk pretreatment for 0.5 h) for 10 h. Each experiment was repeated three times. The data represent the mean ± SD of three independent experiments. AZM represents azithromycin

Annexin V-FITC/PI staining was detected by flow cytometry analysis. Cell death rate in HCT-116 cells was (12.73 ± 1.76)% in the control group, (30.78 ± 8.31)% in the TRAIL group (15.625 nmol/L), (15.73 ± 5.07)% in the azithromycin (50 µmol/L) group, (33.08 ± 7.9)% in the TRAIL + azithromycin (50 µmol/L) group, (24.06 ± 6.16)% in the azithromycin (100 µmol/L) group, and (66.88 ± 11.19)% in the TRAIL + azithromycin (100 µmol/L) group, respectively (Fig. 3a). At 100 µmol/L, azithromycin enhanced the anti-tumor activity of TRAIL more than at 50 µmol/L in HCT-116 cells. Cell death rate in SW480 cells was (13.49 ± 4.63)% in the control group, (56.17 ± 18.51)% in the TRAIL (100 nmol/L) group, (14.20 ± 4.22)% in the azithromycin (50 µmol/L) group, (74.85 ± 7.53)% in the TRAIL + azithromycin (50 µmol/L) group, (21.24 ± 2.76)% in the AZM (100 µmol/L) group, and (89.37 ± 5.07)% in the TRAIL + azithromycin (100 µmol/L) group, respectively. At the concentrations used in the current study, azithromycin induced little cell apoptosis or necrosis, whereas combinations of azithromycin and TRAIL enhanced cell death in both HCT-116 and SW480 cell lines.

The expression of cleaved-PARP was increased in the combination treatment group relative to TRAIL or azithromycin alone (Fig. 3b), which was consistent with the results of flow cytometry analysis. SRB assay showed that zVAD.fmk, a broad-range caspase inhibitor, attenuated the synergistic inhibitory effect of azithromycin and TRAIL on cell proliferation (Fig. 3c). In addition, zVAD.fmk significantly reduced the expression levels of cleaved-PARP (Fig. 3d).

### Synergism between azithromycin and TRAIL in mouse xenograft

**Fig. 4:**
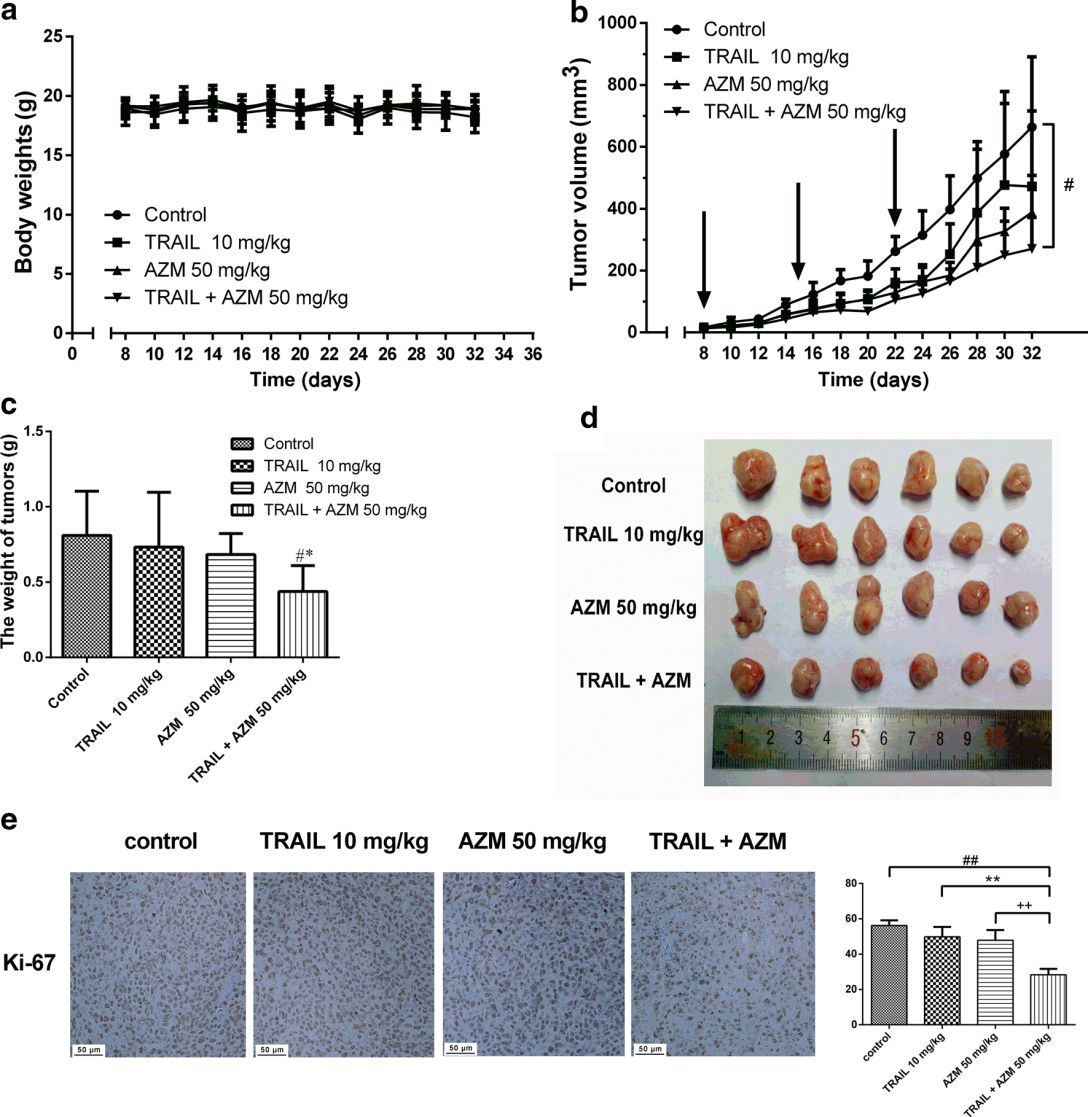
Inhibitory effects of azithromycin and TRAIL on HCT-116 xenografts in nude mice. **a** The body weight of HCT-116 xenograft-bearing nude mice (n = 6). **b** The antitumor effect of azithromycin and/or TRAIL on the growth of HCT-116 xenografts in nude mice (n = 6). ^#^*P *< 0.05 compared with the control. **c** The tumor weight of the four groups. ^#^*P *< 0.05 vs. the control, ^*^P < 0.05 vs. azithromycin. **d** Representative photographs of the excised HCT-116 tumors from four groups. **e** Detection of Ki-67 in HCT-116 xenografts by immunohistochemistry. Cells with brownish yellow particles were considered as Ki-67 positive. Ki-67% was equal to the percentage of Ki-67 positive area and total area. ^##^*P* < 0.01 vs. the control, ^**^P < 0.01 vs. TRAIL. AZM represents azithromycin

No death or significant body weight change was observed in any treatment group (Fig. 4a). Combined treatment with azithromycin and TRAIL significantly inhibited the growth of HCT-116 xenografts (Fig. 4b, c for tumor volume and weight, respectively). The CDI was 0.87. The anti-tumor rate of the combined group was 45.88%. The synergistic effects were also apparent by inspecting the size and morphology of the xenografts (Fig. 4d). Ki-67 was significantly lower in the combination group than either azithromycin or TRAIL alone (Fig. 4e).

### Azithromycin increased the expression of death receptors

**Fig. 5:**
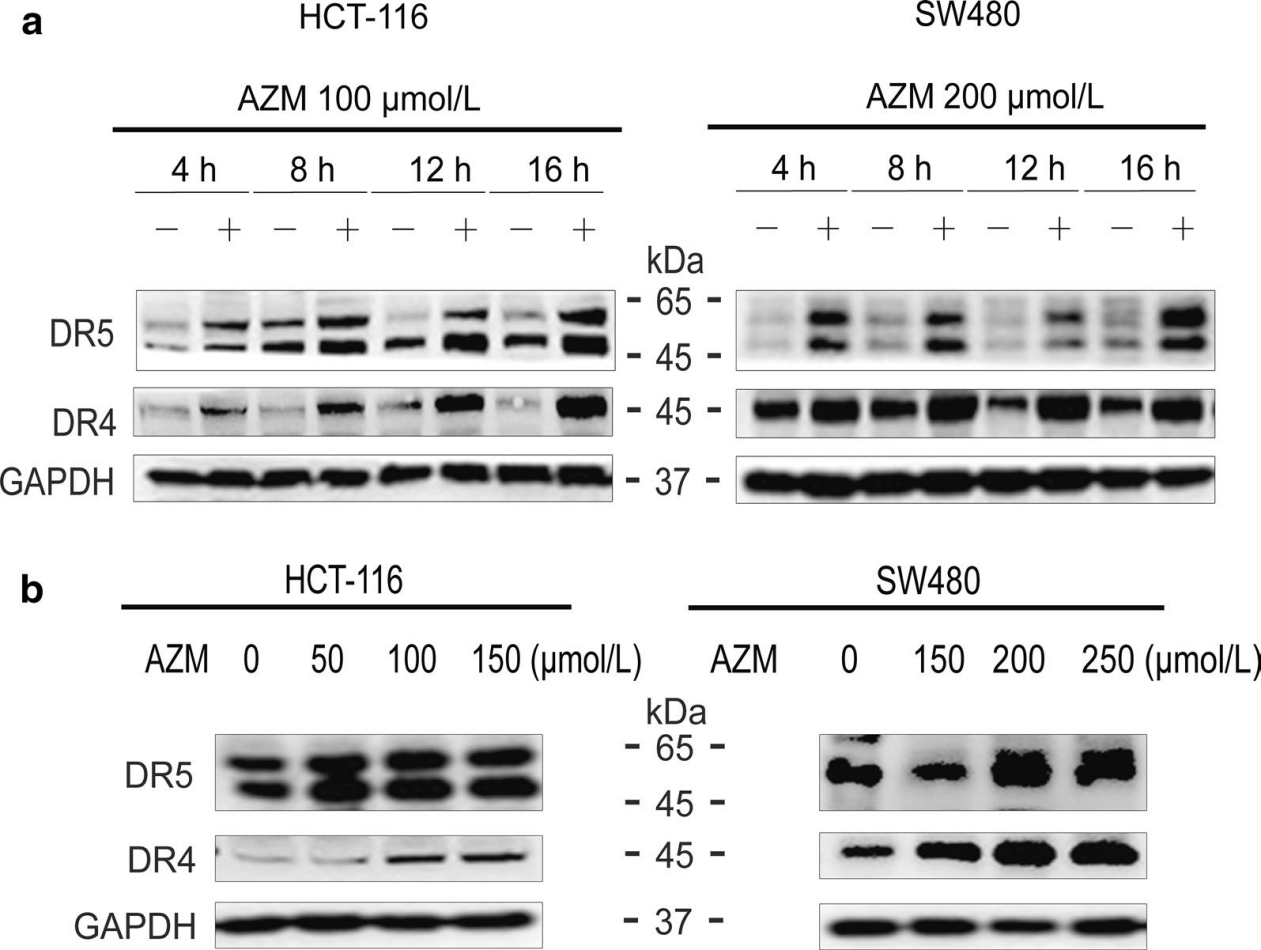
Azithromycin increases the expression of the death receptor proteins. **a** HCT-116 and SW480 cells were treated with azithromycin for the indicated times. **b** Cells were treated with various concentrations of azithromycin for 10 h. The expression levels of proteins were determined using Western blot analysis. The data are shown as the mean ± SD of three independent samples. AZM represents azithromycin

Western blotting was used to examine the changes in DR4 and DR5 protein levels. Azithromycin at the concentration of 100 or 200 µmol/L increased the expression of DR4 and DR5 in 4–16 h in HCT-116 or SW480 cells, respectively (Fig. 5a). Doses of azithromycin at 50–150 or 150–250 µmol/L also elevated the levels of DR4 and DR5 in HCT-116 or SW480 cells, respectively (Fig. 5b).

### Azithromycin blocked autophagy flux

**Fig. 6:**
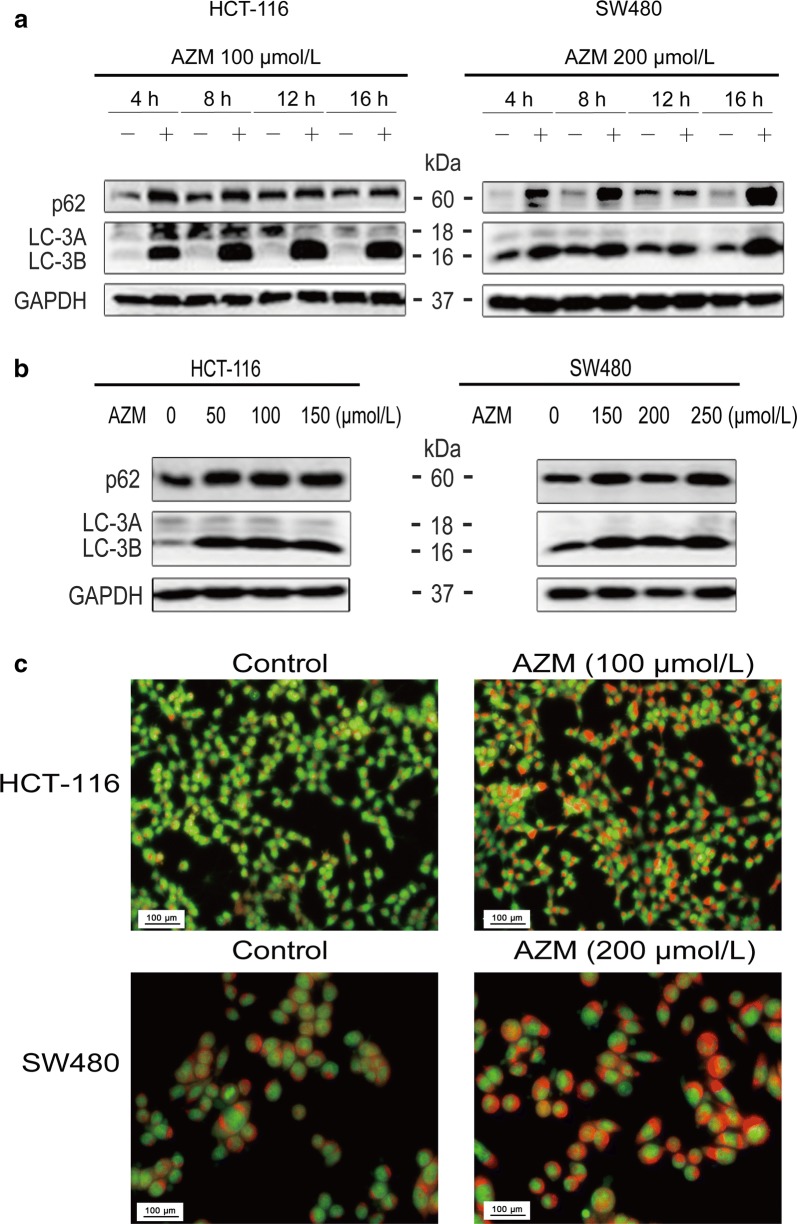
Azithromycin blocks autophagy flux. **a**, **b** HCT-116 and SW480 cells were treated with azithromycin for the indicated times or treated with various concentrations of azithromycin for 10 h. The expression levels of proteins were determined using Western blot analysis. The data are shown as the mean ± SD of three independent experiments. **c** Acridine orange (AO) staining. The green signals represent the nuclei; red signals represent the acidic vesicles, including lysosomes and autophagolysosmes. AZM represents azithromycin

In our experiments, HCT-116 and SW480 cells were treated with azithromycin (100 or 200 µmol/L) or various doses of azithromycin before determination of the expression levels of p62 and LC-3B using Western blot. Briefly, azithromycin increased p62 and LC-3B in both cell lines (Fig. 6a, b). Consistently, AO staining showed increased red fluorescence intensity in both cell lines after treatment with azithromycin for 24 h (Fig. 6c).

### LC-3B-involved autophagy inhibition targeted death receptor 4 and 5 for increment to strengthen TRAIL activity

**Fig. 7:**
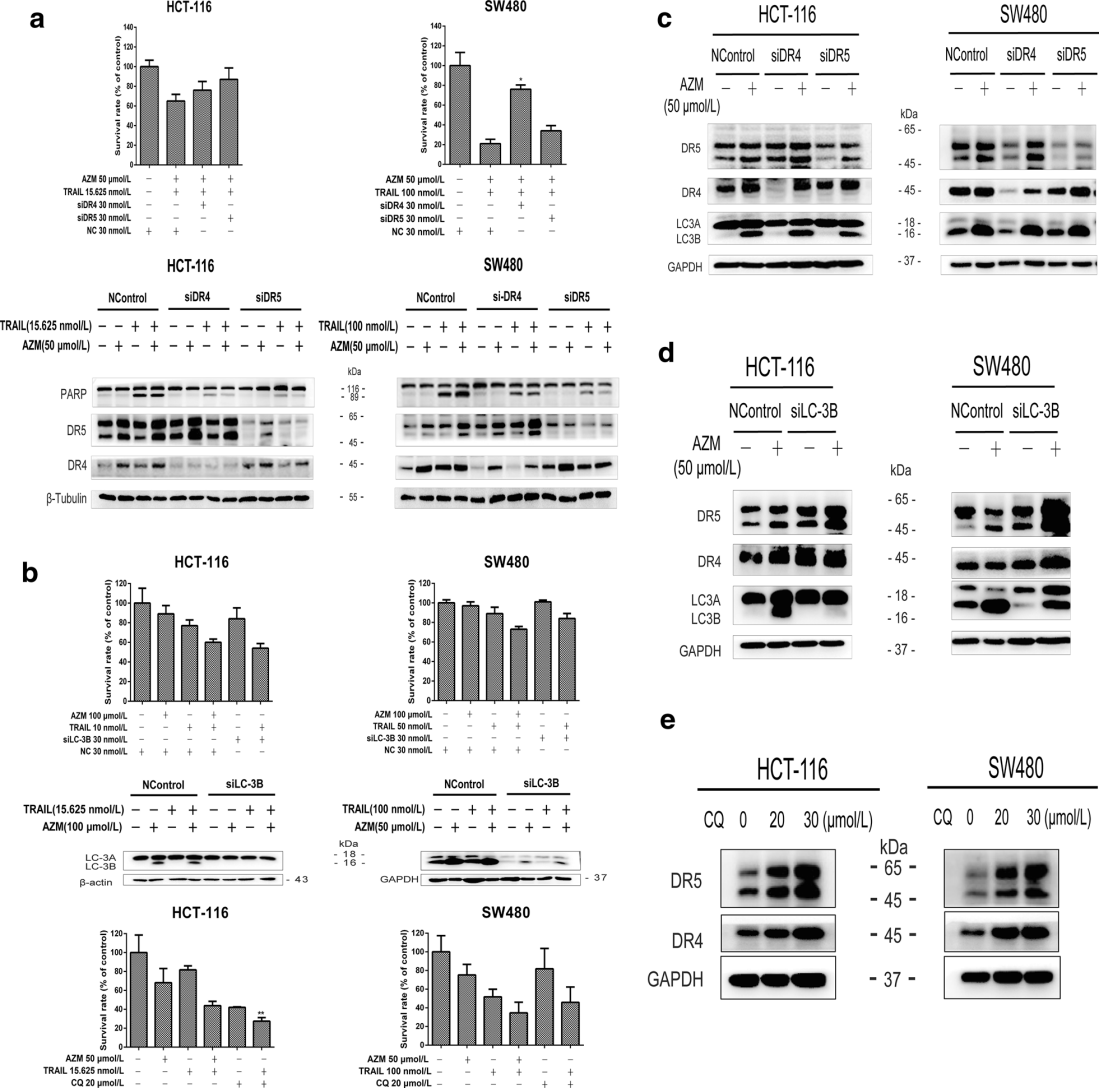
LC-3B-involved autophagy inhibition targets death receptor 4/5 to increase to strengthen TRAIL activity. **a** The viability of SW480 and HCT-116 cells after 24 h pretreatment with 30 nmol/L siRNA (DR4/5) and 48 h-treatment with azithromycin and TRAIL at the indicated concentrations was assessed with the SRB assay. The expression of correlative proteins was assessed by Western blot after 36 h pretreatment with siRNA and 10 h treatment with azithromycin and TRAIL. **b** The treatment time and dose of the LC-3B siRNA-related experiment were the same as in **a**. Both cells were exposed to azithromycin (50 µmol/L), TRAIL (15.625 or 100 nmol/L, respectively) and CQ (20 µmol/L) for 48 h. **c**, **d** HCT-116 and SW480 cells were treated with siRNA for 36–40 h and later exposed to azithromycin and/or TRAIL for 10 h. **e** Cells were treated with CQ (20 and 30 µmol/L) for 10 h. Each experiment was repeated three times. The data represent the mean ± SD of three independent experiments. AZM represents azithromycin

Knocking down of DR4 and DR5 with siRNAs increased survival rates and a decreased cleaved-PARP levels in the cells exposed to azithromycin (50 µmol/L) plus TRAIL (15.625 or 100 nmol/L) (Fig. 7a).

The RIPK1 allosteric inhibitor necrostatin-1 and ROS scavenger *N*-acetylcysteine did not affect the anti-proliferation activity of the combination of azithromycin and TRAIL (Additional file 1: Figure S1A, B), so we next examined whether LC-3B-involved autophagy inhibition is involved in the synergistic antitumor activity between azithromycin and TRAIL. LC-3B siRNA (30 nmol/L) or CQ (20 µmol/L) potentiated the anti-proliferation activity of TRAIL (Fig. 7b). Also, LC-3B level elevated by azithromycin (50 µmol/L) did not change upon knocking down of DR4 or DR5 (Fig. 7c). In contrast, LC-3B siRNA (30 nmol/L) alone or combined with azithromycin (50 µmol/L) increased the expression of DR4 and DR5 (Fig. 7d). Similarly, CQ (20 and 30 µmol/L) elevated DR4 and DR5 levels in a dose-dependent manner (Fig. 7e).

## Discussion

The current study showed that azithromycin could reduce the proliferation of SW480 and HCT-116 colon cancer cells. Varying sensitivity of different colon cancer cells to azithromycin could not be attributed to expression of DR4/5, MAPK and Akt signal pathways-related proteins. TRAIL decreased the viability of SW480 and HCT-116 cells. Most importantly, we showed synergistic inhibitory action between azithromycin and TRAIL on the proliferation in these two cell lines. Our results also suggested that azithromycin could enhance TRAIL-induced apoptosis via a caspase-dependent pathway in SW480 and HCT-116 cells. Experiments in nude mice carrying xenograft confirmed the synergistic action between azithromycin and TRAIL.

Azithromycin has been shown to induce apoptosis and necrosis in HeLa cells [4]. At 50 and 100 µmol/L, azithromycin alone did not induce cell apoptosis or necrosis in the current study. Such a difference could reflect different sensitivity of the cells to azithromycin. Also, the concentration of azithromycin used in our system was lower than needed to induce apoptosis in the previous study. We found increased expression of death receptors DR4 and DR5 upon azithromycin treatment. TRAIL induces apoptosis via specific binding to DR4 and DR5. Moreover, several drugs (i.e., celecoxib, quinacrian, and bleomycin) sensitize certain types of cancer cells to apoptosis induced by TRAIL through increasing the protein levels of DR4 or DR5 [19–22]. We therefore suspect that the death receptor protein levels are implicated in the synergistic action between azithromycin and TRAIL.

Recent studies have shown that azithromycin is an autophagy inhibitor in myeloma and pancreatic cancer cells [6, 7], but whether azithromycin inhibits autophagy in colon cancer cells remains unknown. Autophagy is a lysosomal degradation process that involves two stages: the formation of autophagosomes (the early stage) and sequential fusion with lysosomes to form autolysosomes (the later stage) [23]. Autophagy could be inhibited by disrupting autophagosome formation, for example with 3-MA and ATG 5 siRNA, or by suppressing lysosomal activity, such as with chloroquine (CQ) and bafilomycin A1 [24]. The transformation of LC3 protein, from LC-3A in the cytoplasm to LC-3B that in turn binds the outer membranes, is essential to complete autophagosomes [10]. The p62 protein is a substrate of autophagy, and is widely used together with LC-3B to detect autophagy induction or inhibition. In our study, azithromycin increased the expression of LC-3B and p62 proteins, suggesting that azithromycin produces the anti-tumor effects by blocking autophagy flux.

Our results showed that azithromycin increased the expression of death receptors and blocked autophagy flux in colon cancer cells. The relationship between autophagy inhibition and death receptors-mediated cell death requires further investigation. Knockdown of DR4 and DR5 with siRNA increased survival rate of colon cancer cells and decreased cleaved-PARP. All together, these findings suggest that azithromycin treatment enhanced TRAIL-induced apoptosis via the up-regulation of DR4 and DR5 in colon cancer cells. A previous study showed that DR4 and DR5 co-localize with LC-3B on the surface of autophagosomes, which result in the decrease of the surface expression of DR4/5 and eventually induce TRAIL resistance in breast cancer cells [10]. Interestingly, in our experimental system, azithromycin and CQ simultaneously increased the expression of DR4, DR5 and LC-3B in colon cancer cells, despite of enhancing the anti-proliferation effects of TRAIL. In addition, the knockdown of DR4 or DR5 did not affect the expression of LC-3B. In contrast, the reduction in the LC-3B protein level increased the expression of DR4 and DR5 to some extent, and LC-3B siRNA also raised the anti-survival effect of TRAIL. Notably, not all autophagy inhibitors could increase the expression of DR4/5 protein. For instance, it has been reported that 3-MA decreased DR4/5 proteins levels in colon cancer cells [25]. Thus we speculate that autophagy inhibition in early vs. late stages could lead to opposite results. Accordingly, azithromycin, CQ and LC-3B cooperate with TRAIL to produce antitumor effects via the induction of LC-3B-involved autophagy inhibition to up-regulate DR4/5 proteins.

In addition to apoptosis induction, TRAIL causes RIP1-dependent necroptosis [26–28]. By the RIPK1 allosteric inhibitor necrostatin-1 did not affect the anti-proliferation activity of the combination of azithromycin and TRAIL in our experimental system. Several previous studies have noted that the up-regulation of death receptors is associated with the activation of reactive oxygen species (ROS) [20, 29, 30]. The results obtained with the ROS scavenger *N*-acetylcysteine in the current study suggest that the ROS pathway is not involved in the mechanism of the synergistic antitumor effect between azithromycin and TRAIL.

**Fig. 8:**
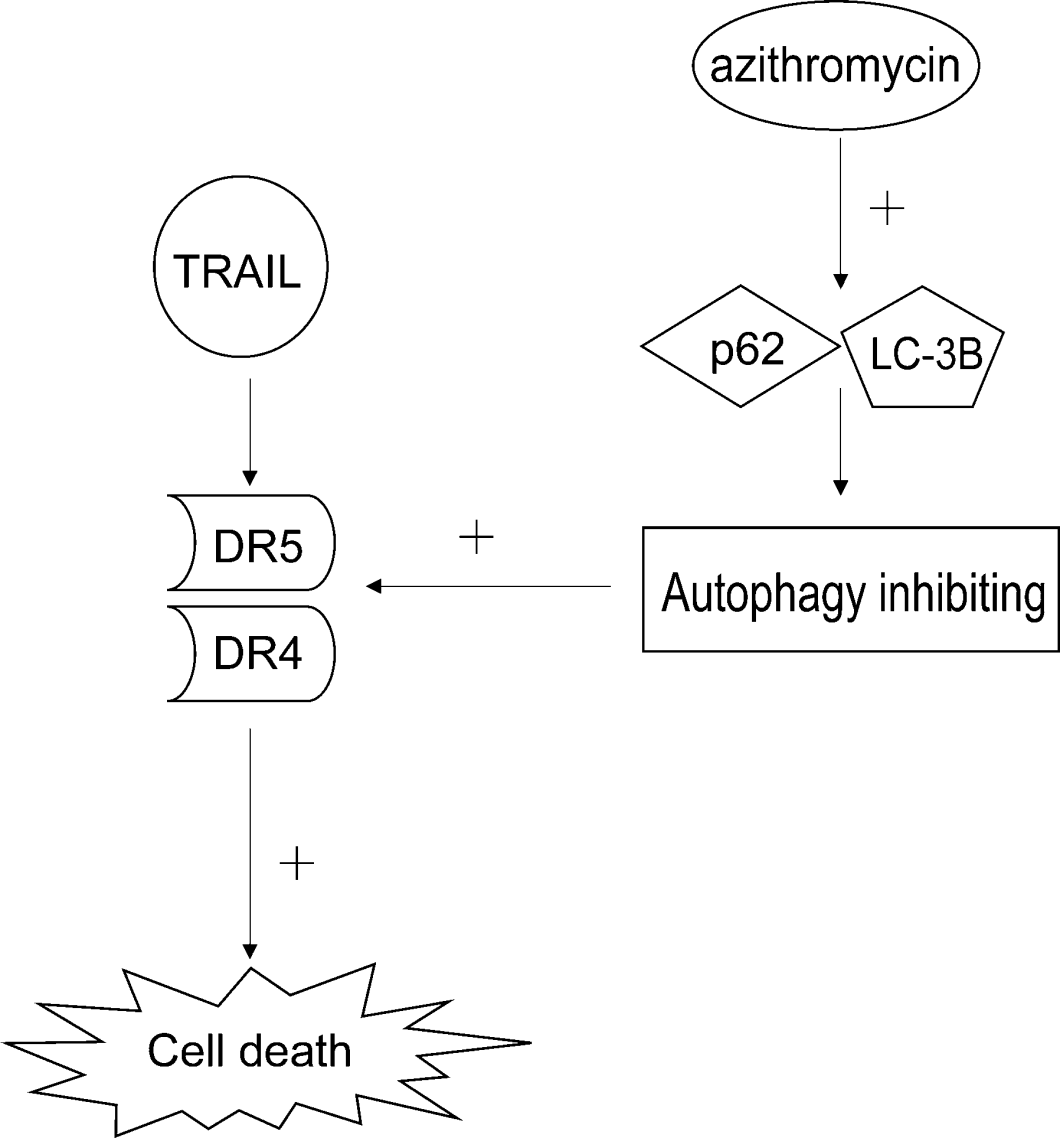
Schematic presentation of the synergistic inhibitory mechanism. In colon cancer cells, azithromycin inhibits autophagy via up-regulation of p62 and LC-3B, resulting in an increase in DR4/5, which enhances the antitumor activity of TRAIL, and ultimately augment colon cancer cell death

There are some limitations in the current study. For example, in animal experiments, the standard deviation in tumor size was quite large. Also, participation of signaling pathways other than autophagy flux was not examined. Regardless of these limitations, the synergistic action between azithromycin and TRAIL is apparent. A schematic diagram of the working hypothesis is shown in Fig. 8.

## Conclusions

Azithromycin could enhance the anticancer activity of TRAIL via LC-3B-involved autophagy inhibition to up-regulate DR4/5 in colon cancer cells both in vitro and in vivo. These results encourage further studies to explore using azithromycin in the treatment of colon cancer.

## Additional file

**Additional file 1: Figure S1.** The ROS pathway and necroptosis are not involved in the combined antitumor effect of azithromycin and TRAIL. (A) NAC did not affect the synergistic inhibitory effect of azithromycin and TRAIL. The viability of HCT-116 and SW480 cells after 0.5 h-pretreatment with NAC (5 mmol/L) followed by a 48 h treatment with azithromycin (50 µmol/L) and TRAIL (15.625 or 100 nmol/L, respectively) was assessed using the SRB assay. (B) Necrostatin-1 did not affect the combined antitumor effect of azithromycin and TRAIL in the colon cells. Both cells were treated with azithromycin (100 µmol/L), TRAIL (100 nmol/L) and necrostatin-1 (25, 50, 75 and 100 µmol/L) for 48 h. Values represent the mean ± SD of three independent experiments. AZM represents azithromycin.

## Abbreviations

3-MA: 3-methyl adenine
AO: acridine orange
CQ: chloroquine
DR4/5: death receptor 4/5
LC-3B: microtubule associated protein 1 light chain 3 beta
NAC: *N*-acetylcysteine
PARP: poly-ADP-ribose polymerase
RAPA: rapamycin
RIPK1: receptor interacting protein kinase 1
ROS: reactive oxygen species
SRB: sulforhodamine B
TCA: trichloroacetic acid
TRAIL: tumor necrosis factor-related apoptosis-inducing ligand
zVAD.fmk: benzyloxycarbonyl-Val-Ala-Asp (OMe) fluoromethylketone
